# Comparative analysis of the complete mitochondrial genomes of three rockfishes (Scorpaeniformes, *Sebastiscus*) and insights into the phylogenetic relationships of Sebastidae

**DOI:** 10.1042/BSR20203379

**Published:** 2020-12-14

**Authors:** Chenghao Jia, Xiumei Zhang, Shengyong Xu, Tianyan Yang, Takashi Yanagimoto, Tianxiang Gao

**Affiliations:** 1 Key Laboratory of Mariculture, Ministry of Education, Ocean University of China, Qingdao, Shandong, China; 2 Function Laboratory for Marine, Fisheries Science and Food Production Processes, Qingdao National Laboratory for Marine Science and Technology, Qingdao, Shandong, China; 3 Fishery College, Zhejiang Ocean University, Zhoushan, Zhejiang, China; 4 National Research Institute of Fisheries Science, Japan Fisheries Research and Education Agency, Yokohama, Kanagawa, Japan

**Keywords:** mitochondrial genome, phylogenetic analysis, Sebastidae, Sebastiscus

## Abstract

Mitochondrial genome is a powerful molecule marker to provide information for phylogenetic relationships and revealing molecular evolution in ichthyological studies. *Sebastiscus* species, a marine rockfish, are of essential economic value. However, the taxonomic status and phylogenetic relationships of Sebastidae have been controversial so far. Here, the mitochondrial genomes (mitogenomes) of three species, *S. tertius, S. albofasciatus*, and *S. marmoratus*, were systemically investigated. The lengths of the mitogenomes’ sequences of *S. tertius, S. albofasciatus*, and *S. marmoratus* were 16910, 17056, and 17580 bp, respectively. It contained 13 protein-coding genes (PCGs), two ribosomal RNAs (rRNAs), 22 transfer RNA (tRNA) genes, and one identical control region (D-loop) among the three species. The genetic distance and *K*_a_/*K*_s_ ratio analyses indicated 13 PCGs were suffering purifying selection and the selection pressures were different from certain deep-sea fishes, which were most likely due to the difference in their living environment. The phylogenetic tree was constructed by Bayesian Inference (BI) and Maximum Likelihood (ML). Most interestingly, the results indicated that Sebastidae and Scorpaenidae were grouped into a separate branch, so the taxonomic status of Sebastidae should be classified into subfamily Sebastinae. Our results may lead to a taxonomic revision of Scorpaenoidei.

## Introduction

The family Sebastidae belongs to Scorpaeniformes, commonly known as rockfishes or false kelpfishes, comprises 7 genera and approximately 133 species, and it is a diverse and economically important group distributed in Atlantic, Indian, and Pacific Oceans [1]. Sebastidae has become one of the most important marine fishes due to its huge economic value and high biodiversity. However, there is still no consensus about the taxonomic status of Sebastidae up to now. Some authors considered Sebastidae as a family of the order Scorpaeniformes [2–6]. By contrast, some authorities recognized it as a subfamily of Scorpaenidae [7–12]. Besides, the databases that are widely referenced also showed different results, like FishBase (https://www.fishbase.se) acknowledged the validity of Sebastidae, but Integrated Taxonomic Information System (ITIS) (https://www.itis.gov) is opposed to this opinion. Before the conclusion, it will be unified use of the appellation ‘Sebastidae’ in the present study.

The genus *Sebastiscus* is mainly distributed in the western Pacific [13,14]. Despite their diversity, abundance, and economic importance, our understanding of the relationships within the genus remains limited. It was classified as part of the genus *Sebastes* until 1904 when Jordan and Starks identified it as a separate genus by recognizing the development of the air bladders [13]. Besides, Matsubara used the structure of the suborbital bone and the number of vertebrae as the diagnosis traits of *Sebastiscus* [15,16]. Although Barsukov and Chen classified *Sebastiscus* as a subgenus of *Sebastes*, it is commonly regarded as an independent genus at present [17–21]. Three valid species and a newly reported species are included in genus *Sebastiscus*: *S. albofasciatus* [22], *S. marmoratus* [23], *S. tertius* [17], and *S. vibrantus* [6]. It is worth noting that *S. marmoratus* is widely distributed in the northwestern Pacific Ocean and the remaining species are likely to be confined to the warm waters of East Asia and Indonesia [6,24].

Mitochondrial genomes (mitogenomes) have become a powerful molecule marker of species classification, population genetics, molecular systematic geography, molecular ecology, and other fields [25–29]. Small size, maternal inheritance, compact gene arrangement, high conservatism, and simple structure are the main features of mitochondrial genomes [30–32]. The structure, characteristics, and properties of the mitochondrial genomes of fish have been studied more and more widely since Johansen et al. completed the complete sequence determination of the mitochondrial genome of *Gadus morhua* [33].

From the above, there have been so many meaningful achievements about phylogenetic analyses and fish taxa through research of mtDNA. However, there exists some limitations in short mitochondrial gene fragments in discussing and resolving more complicated phylogenetic relationships in many fish lineages [34]. For these limitations, the longer DNA sequences liked complete mitochondrial genomes which have additional informative sites will have better ways to solve these higher level relationships and deeper branches thoroughly [35]. Therefore, the mitochondrial genomes applied in this study may help recognize the evolutionary and relationships of the family Sebastidae and verify the accuracy of traditional taxonomy.

In the present study, we sequenced and annotated three complete mitochondrial genomes of *Sebastiscus* species (*S. tertius, S. albofasciatus*, and *S. marmoratus*) and compared them with each other. The characteristics were described to evaluate the variation and conservation in the mitochondrial genome among *Sebastiscus* species. The relative synonymous codon usage (RSCU) and AT skew value of protein-coding genes (PCGs) were analyzed to better understand the functional inference of related genes. Moreover, the phylogenetic analyses among *Sebastiscus* species were performed and related species in Scorpaenoidei to preferably discuss the taxonomic status of Sebastidae. All information reported in this article will help supplement and enhance the limited molecular data available for *Sebastiscus* species, and provide the essential evidence for the taxonomic status of Sebastidae.

## Materials and methods

### Sampling and DNA extraction

The sample of rockfishes were collected using hook-and-line fishing from the coastal waters of Zhoushan in China (coordinates: 30.0607°N, 122.3546°E) during April 2018, coastal waters of Qingdao in China (coordinates: 35.7607°N, 120.2016°E) during May 2019, and coastal waters of Kozagawa in Japan (coordinates: 33.4145°N, 135.7524°E) during June 2019, respectively. All samples were identified based on morphological characteristics (Katoh and Tokimura (2001) [10]; Morishita et al. (2018) [6]) and stored at −20°C for further study. Muscle tissue was taken from the tails of each sample and digested using proteinase K (Merck, Germany). Genomic DNA was isolated following a standard phenol–chloroform method and detected by 2.0% agarose electrophoresis. DNA precipitation was dissolved in double-distilled water and stored at 4°C after concentration quantification. The study protocol was approved by the Experimental Animal Ethics Committee of the Ocean University of China.

### PCR amplification and sequencing

Three *Sebastiscus* mitogenomes were amplified with 34 pairs of *Sebastiscus*-specific universal primer sets (Supplementary Table S1) and 4 pairs of specific primer sets (Supplementary Table S2) which were designed based upon the mitochondrial genome sequence of *S. marmoratus* (accession no. KF667491) by Primer Premier 6.0 [36]. We used the normal PCR method with Takara Taq DNA polymerase (Takara, China) in a 25-µl reaction volume including 17.5 µl of ultrapure water, 2.5 µl of 10× PCR buffer, 2 µl of dNTPs, 1 µl of each primer (5 µmol/l), 0.15 µl of Taq polymerase, and 1 µl of DNA template. PCR amplification was carried out in a Biometra thermal cycler (Göttingen, Germany) under the following conditions: 5 min initial denaturation at 95°C, and 35 cycles of 45 s at 94°C for denaturation, 45 s at 53°C for annealing, and 45 s at 72°C for an extension, and a final extension at 72°C for 10 min. All PCR products were sequenced in both directions using the primer-walking method by Qingdao Qingke Biotechnology Co. Ltd. (Qingdao, China).

### Mitogenome annotation and sequence analyses

SeqMan (DNAStar, U.S.A.) software was used to manually correct and compare the contiguous sequence fragments, then splice the complete mitogenome sequence and calculate the full length of mitogenome. The starting and ending positions of each gene were determined by comparing the full mitogenome sequence of *S. marmoratus* (Accession no. KF667491) published in GenBank (https://www.ncbi.nlm.nih.gov/genbank/). Online software tRNAscan-SE2.0 (https://lowelab.ucsc.edu/tRNAs can-SE) was used to identify the transfer RNA (tRNA) gene and predict the secondary structure diagram of tRNA [37]. The online software Tandem Repeats Finder [38] was used to search and analyze the tandem repeats in the control region. The O_L_ and tandem repeat structures were simulated and drawn by RNAstructure6.2 software. All complete mitogenomes were preliminarily annotated and drawn the mitochondrial genome map by Mitofish (https://mitofish.aori.u-tokyo.ac.jp) [39,40].

The composition of complete mitochondrial genome sequence and each segment (including the non-coding region, ribosomal RNA (rRNA), tRNA, and the PCGs) were calculated by MEGA5.0 software [41], and the PCG codon and base content were determined. Composition skewness of each segment was calculated by the following formulas: AT-skew = (A − T)/(A + T); GC-skew = (G − C)/(G + C).

### Phylogenetic analyses

In order to discuss the phylogenetic relationships of Sebastidae and explore the taxonomic status of *Sebastiscus* in Scorpaenoidei, mitogenomes of 33 previously sequenced Scorpaenoidei and 2 previously sequenced Gobiidae species (with the latter as the outgroup taxon; Table 1) were used in the phylogenetic analyses. We used the nucleotide sequences of the 13 PCGs as the dataset to construct the phylogenetic tree. Sequences were aligned using SeqMan from DNAStar software (U.S.A.). The optimal model for nucleotide sequences was estimated by MEGA 5.0 (Tamura et al. 2011). TN93 + G + I captured the minimum values of Bayesian Information Criterion (BIC) and it was considered to be the best model for phylogenetic tree construction. The Maximum Likelihood (ML) phylogenetic tree was constructed by MEGA 5.0 software with 1000 replicates of bootstrap and the Bayesian Inference (BI) analysis was inferred by the software of MrBayes 3.2.6 based on 10000000 generations [42]. The divergence time was predicted by MEGA 10 [43] with the ML method of RelTime. The calibration of divergence times was obtained from online Time Tree database (http://www.timetree.org/) [44].

**Table 1:** Information of the complete mitogenome sequences cited in this study

Family (Latin name)	Species (Latin name)	GenBank number	Length (bp)
Sebastidae	*Sebastes oblongus*	KF836441	16396
	*Sebastes fasciatus*	KX897946	16400
	*Sebastes thompsoni*	KJ834064	16405
	*Sebastes trivittatus*	KJ834062	16409
	*Sebastes pachycephalus*	KF836442	16415
	*Sebastes longispinis*	KJ834061	16445
	*Sebastes taczanowskii*	KJ525744	16452
	*Sebastes hubbsi*	KJ525745	16453
	*Sebastes vulpes*	KJ525743	16462
	*Sebastes owstoni*	KJ834063	16465
	*Sebastes inermis*	KF725093	16504
	*Sebates schlegelii*	AY491978	16525
	*Sebastes minor*	MH378782	16850
	*Sebastes rubrivinctus*	MH378777	16896
	*Sebastes nigrocinctus*	MH378778	16922
	*Sebastes aleutianus*	MH378781	17038
	*Sebastes steindachneri*	MH378779	17109
	*Sebastes koreanus*	KJ775792	17606
	*Helicolenus avius*	NC020349	16651
	*Helicolenus hilgendorfi*	AP002948	16728
Scorpaenidae	*Pterois miles*	LK022697	16497
	*Pterois volitans*	KJ739816	16500
	*Parapterois heterura*	LC493917	16475
	*Scorpaenopsis ramaraoi*	LC493915	16972
	*Scorpaenopsis cirrosa*	KR701907	16966
Peristediidae	*Satyrichthys amiscus*	AP004441	16526
Triglidae	*Chelidonichthys kumu*	KY379222	16495
	*Pterygotrigla hemisticta*	LC493913	16499
	*Pterygotrigla ryukyuensis*	LC495487	16508
	*Lepidotrigla kanagashira*	MK784116	16504
	*Lepidotrigla guentheri*	LC493914	16509
	*Lepidotrigla hime*	MN104592	16606
	*Lepidotrigla microptera*	KY012348	16610
Gobiidae	*Parapocryptes serperaster*	KT965855	17243
	*Boleophthalmus pectinirostris*	NC016195	17111

## Results and discussion

### Mitogenome organization and composition

**Figure 1:**
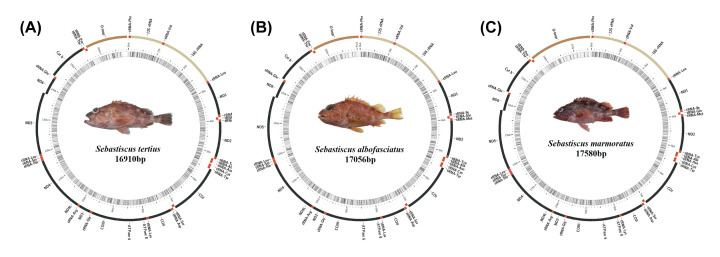
Mitochondrial genome maps of *S. tertius* (A) *S. albofasciatus* (B) and *S. marmoratus* (C) The innermost circle of the images represents GC% per every 5 bp of the mitogenome; the darker lines are, the higher their GC% are.

**Figure 2:**
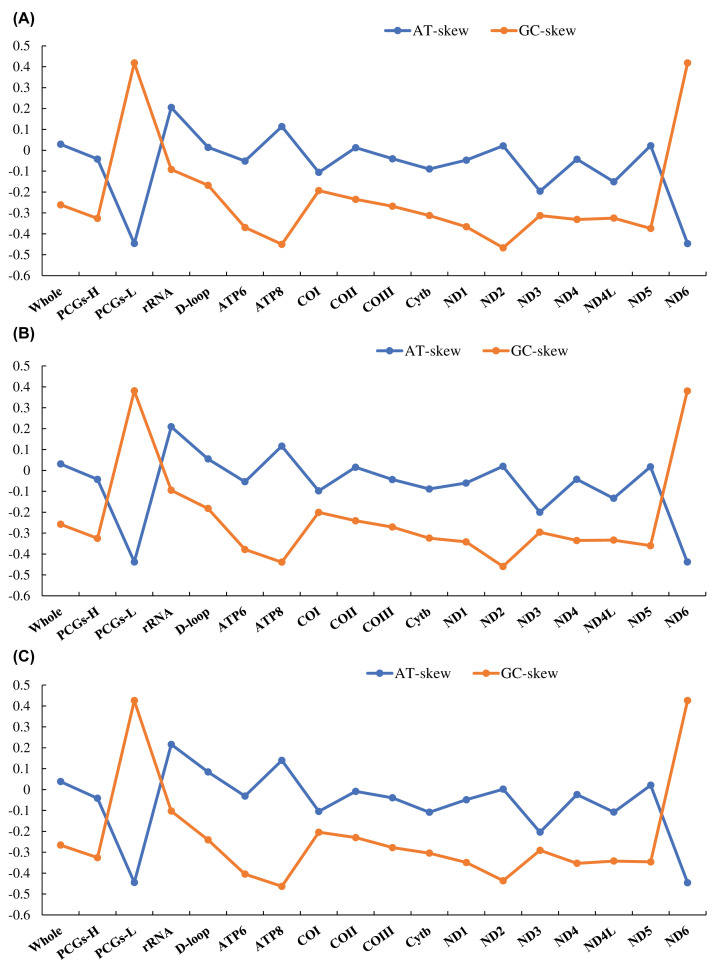
The nucleotide skewness of three species of *Sebastisucs* (**A**) *S. tertius*; (**B**) *S. albofasciatus*; (**C**) *S. marmoratus*. The incomplete T-/TA- of the stop codon is not included.

The complete mitochondrial genomes of *S. albofasciatus* (Accession no. MT117230), *S. tertius* (Accession no. MT117231), and *S. marmoratus* (Accession no. MT789709) in GenBank were 17056, 16910, and 17580 bp in length, respectively (Figure 1). The size variation of the three mitogenomes was mainly caused by the differences in the lengths of the non-coding regions. Of all 36 sequenced Scorpaenoidei mitogenomes, the length of the mitogenome of *Sebastes koreanus* (17606 bp) was the longest, whereas that of *Sebastes oblongus* (16396 bp) was the shortest. The mitogenome lengths of five Scorpaenoidei species were longer (>17000 bp) because of a longer control region (>1300 bp). The mitogenome of *S. albofasciatus* contained the typical 37 genes (13 PCGs, 22 tRNAs, and 2 rRNAs), 1 control region, and 1 OL. The mitogenome of *S. tertius* and *S. marmoratus* had the same composition (Table 2). Most mitochondrial genes were encoded on the H-strand, except for ND6 and eight tRNA (Glu, Ala, Asn, Cys, Tyr, Ser-UCN, Gln, and Pro) genes that were encoded on the L-strand. The nucleotide composition of *S. albofasciatus, S. tertius*, and *S. marmoratus* mitogenomes had a higher A+T bias of 55.15, 55.04, and 55.30%, respectively, and both showed positive AT-skew and negative GC-skew (Figure 2 and Supplementary Table S3).

**Table 2:** Summary of gene/element feature of *S. tertius* (ST), *S. albofasciatus* (SA) and *S. marmoratus* (SM)

Gene/Region	Position start			Position end			Size (bp)			Intervening spacer (bp)*			Amino acid			Initial codon			Terminal codon			Strand	Letter code
	ST	SA	SM	ST	SA	SM	ST	SA	SM	ST	SA	SM	ST	SA	SM	ST	SA	SM	ST	SA	SM		
tRNA^Phe^	1	1	1	68	68	68	68	68	68	0	0	0										H	F
12S rRNA	69	69	69	1015	1014	1014	947	946	946	0	0	0										H	
tRNA^Val^	1016	1015	1015	1087	1086	1086	72	72	72	0	0	0										H	V
16S rRNA	1088	1087	1087	2779	2778	2778	1692	1692	1692	0	0	0										H	
tRNA^Leu(UUR)^	2780	2779	2779	2853	2852	2852	74	74	74	0	0	0										H	L1
ND1	2854	2853	2853	3828	3827	3827	975	975	975	3	5	3	324	324	324	ATG	ATG	ATG	TAG	TAA	TAA	H	
tRNA^Ile^	3832	3833	3831	3903	3902	3902	72	70	72	−2	−1	−2										H	I
tRNA^Gln^	3902	3902	3901	3972	3972	3972	71	71	72	−1	−1	−2										L	Q
tRNA^Met^	3972	3972	3971	4042	4042	4041	71	71	71	0	0	0										H	M
ND2	4043	4043	4042	5088	5088	5087	1046	1046	1046	0	0	0	348	348	348	ATG	ATG	ATG	TA	TA	TA	H	
tRNA^Trp^	5089	5089	5088	5159	5160	5158	71	71	71	1	1	1										H	W
tRNA^Ala^	5161	5162	5160	5229	5230	5228	69	69	69	1	1	1										L	A
tRNA^Asn^	5231	5232	5230	5303	5304	5302	73	73	73	1	1	0										L	N
O_L_	5305	5306	5303	5341	5342	5340	37	37	38	−3	−3	−4										H	
tRNA^Cys^	5339	5340	5337	5405	5406	5403	67	65	67	2	2	2										L	C
tRNA^Tyr^	5408	5409	5406	5478	5479	5476	69	71	71	1	1	1										L	Y
COI	5480	5481	5478	7030	7031	7028	1551	1551	1551	0	0	0	516	516	516	GTG	GTG	GTG	TAA	TAA	TAA	H	
tRNA^Ser(UCN)^	7031	7032	7029	7101	7102	7099	71	69	71	3	3	3										L	S
tRNA^Asp^	7105	7106	7103	7177	7178	7175	73	73	73	6	6	6										H	D
COII	7184	7185	7182	7874	7875	7872	691	691	691	0	0	0	230	230	230	ATG	ATG	ATG	T	T	T	H	
tRNA^Lys^	7875	7876	7873	7948	7949	7946	74	74	74	1	1	1										H	K
ATPase8	7950	7951	7948	8117	8118	8115	168	168	168	−10	−10	−10	55	55	55	ATG	ATG	ATG	TAA	TAA	TAA	H	
ATPase6	8108	8109	8106	8790	8791	8788	683	683	683	0	0	0	227	227	227	ATG	ATG	ATG	TA	TA	TA	H	
COIII	8791	8792	8789	9575	9576	9573	785	785	785	0	0	0	261	261	261	ATG	ATG	ATG	TA	TA	TA	H	
tRNA^Gly^	9576	9577	9574	9647	9648	9645	72	72	72	0	0	0										H	G
ND3	9648	9649	9646	9996	9997	9994	349	349	349	0	0	0	116	116	116	ATG	ATG	ATG	T	T	T	H	
tRNA^Arg^	9997	9998	9995	10065	10066	10063	69	69	69	0	0	0										H	R
ND4L	10066	10067	10064	10362	10363	10360	297	297	297	−7	−7	−7	98	98	98	ATG	ATG	ATG	TAA	TAA	TAA	H	
ND4	10356	10357	10354	11736	11737	11734	1381	1381	1381	0	0	0	460	460	460	ATG	ATG	ATG	T	T	T	H	
tRNA^His^	11737	11738	11735	11805	11806	11803	69	69	69	0	0	0										H	H
tRNA^Ser(AGY)^	11806	11807	11804	11873	11874	11871	68	72	68	4	4	4										H	S
tRNA^Leu(CUN)^	11878	11879	11876	11950	11951	11948	73	73	73	0	0	0										H	L2
ND5	11951	11952	11949	13789	13790	13787	1839	1839	1839	−4	−4	−4	612	612	612	ATG	ATG	ATG	TAA	TAA	TAA	H	
ND6	13786	13787	13784	14307	14308	14305	522	522	522	0	0	0	173	173	173	ATG	ATG	ATG	TAG	TAG	TAG	L	
tRNA^Glu^	14308	14309	14306	14376	14377	14374	69	69	69	6	6	6										L	E
Cyt *b*	14383	14384	14381	15523	15524	15521	1141	1141	1141	0	0	0	380	380	380	ATG	ATG	ATG	T	T	T	H	
tRNA^Thr^	15524	15525	15522	15595	15596	15593	72	73	72	−1	−1	−1										H	T
tRNA^Pro^	15595	15596	15593	15664	15665	15662	70	70	70	0	0	0										L	P
D-loop	15665	15666	15663	16910	17056	17580	1246	1391	1918													H	

Intervening spacer* (bp): positive values indicate the interval sequence of adjacent genes, and negative values indicate the overlapping of adjacent genes. H represents heavy strand and L represents light strand.

### PCGs and codon usages

All the 13 PCGs in the mitogenomes of the three rockfishes were similar to those of other vertebrates. Twelve PCGs (*ND1, ND2, COI, COII, ATP8, ATP6, COIII, ND3, ND4L, ND4, ND5*, and *CYTB*) were coded on the heavy strand (H-strand) and the remaining one (ND6) was coded on the light strand (L-strand). The length, codon usage, and A+T content of PCGs in the *S. albofasciatus, S. tertius*, and *S. marmoratus* mitogenomes were nearly identical. Among the three mitogenomes, all the 13 PCGs of *Sebastiscus* species encoded 3800 amino acids in total. All the PCGs used the initiation codon ATG except COI used GTG. ATG is an accepted conventional initiation codon for many Osteichthyes mitogenomes including among Scorpaeniformes fishes [45,46]. At the same time, GTG was commonly used as the initiation codon of COI in many other Osteichthyes mitogenomes [47–51]. All three mitogenomes have the same termination codon for 11 PCGs (*ND2, COII, ATP8, ATP6, COIII, ND3, ND4L, ND4, ND5, ND6*, and *CYTB*). TAA were commonly used as the termination codons, although the incomplete termination codons T or TA were found in ND2, COII, ATP6, COIII, ND3, ND4, and CYTB in all three mitogenomes. The incomplete termination codon has also been found in all other sequenced Osteichthyes species [48–52]. It has been confirmed that the incomplete termination codons could act as the complete functional termination codons in polyadenylation processes and polycistronic transcription cleavage [29,51,53]. TAG was used as the termination codon of ND6 in the three *Sebastiscus* mitogenomes. Additionally, in the *S. tertius* mitogenome, ND1 used TAG as the termination codon, too. Although TAG is the typical termination codon in many mitogenomes, it is not used frequently, due to the high percentage of AT nucleotide use by the PCGs [32].

**Figure 3:**
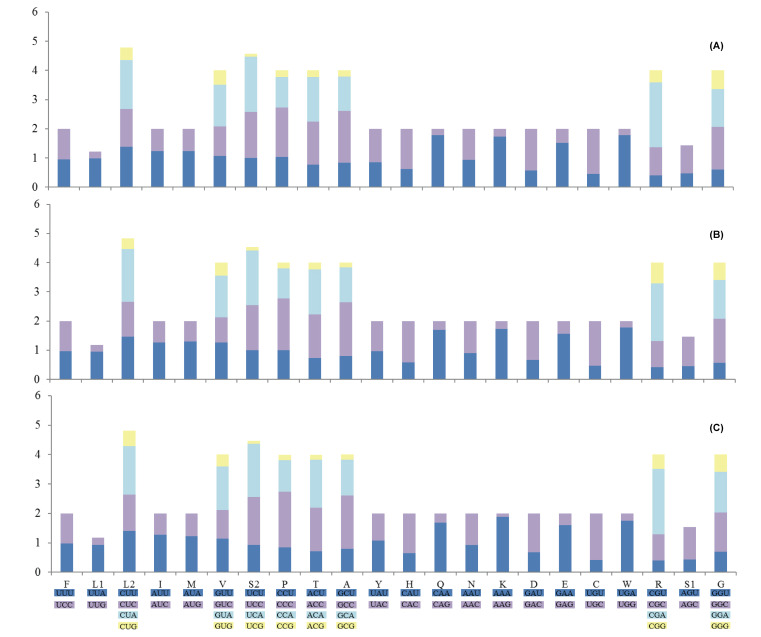
The RSCU in three *Sebastiscus* mitogenomes The RSCU of the mitogenome in *S. tertius* (**A**), *S. albofasciatus* (**B**), and *S. marmoratus* (**C**). Codon families are labeled on the x-axis. The termination codon is not given.

The average AT contents of the 13 PCGs in *S. albofasciatus* and *S. tertius* were 54.50 and 54.40%, respectively, and both were similar values to that of *S. marmoratus* (54.54%). The PCGs encoded by the H-strand displayed T-skews (T > A) and C-skews (C > G) whereas the L-strand displayed T-skews and G-skews (G >C). We calculated the RSCU of the three *Sebastiscus* species mitogenomes (Figure 3 and Supplementary Table S4) and the result showed that the frequency of using NNA and NNC (N represents A, T, C, G) were higher than NNT and NNG in *S. albofasciatus, S. tertius*, and *S. marmoratus.* The most frequent amino acids in the coding sequences of *S. albofasciatus, S. tertius*, and *S. marmoratus* mitochondrial proteins were Leu (CUN), Ala, and Thr (>290) (Supplementary Figure S1). These three amino acids were also frequently used in other Osteichthyes mitogenomes [48–51]. Moreover, the minimally used amino acid in the three mitogenomes was Cys (<30).

### Genetic distance and evolution rates of PCGs

**Figure 4:**
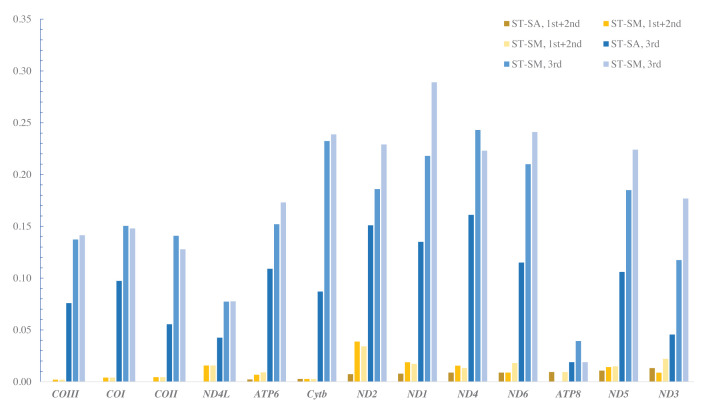
The pair genetic distances of 13 PCGs among *S. tertius* (ST), *S. albofasciatus* (SA), and *S. marmoratus* (SM) The values were calculated based on the first and second nucleotide position, and on the third nucleotide position, respectively.

The genetic distance could be used to evaluate different mutation pressures among genes [54]. The pairwise genetic distances (p-distance) were calculated to reveal the sequence conservation and divergence of the PCGs among the *Sebastiscus* species (Figure 4). The genetic distance at the third nucleotide position was obviously higher than the first and second nucleotide position, indicating that the evolution of the third position was faster than the first and the second. The highest p-distance was found in ND1 (0.289) and ND4 (0.243, 0.161) at the third nucleotide of codons, while explored in ND2 (0.039, 0.034) and ND3 (0.013) base on the first and second nucleotide position. The COI-III and ATP8 genes had the low genetic distance in both first + second and third analysis. ND1, ND2, ND3, and ND4 genes might have high evolutionary rates among the three species, while COI-III and ATP8 were low.

**Figure 5:**
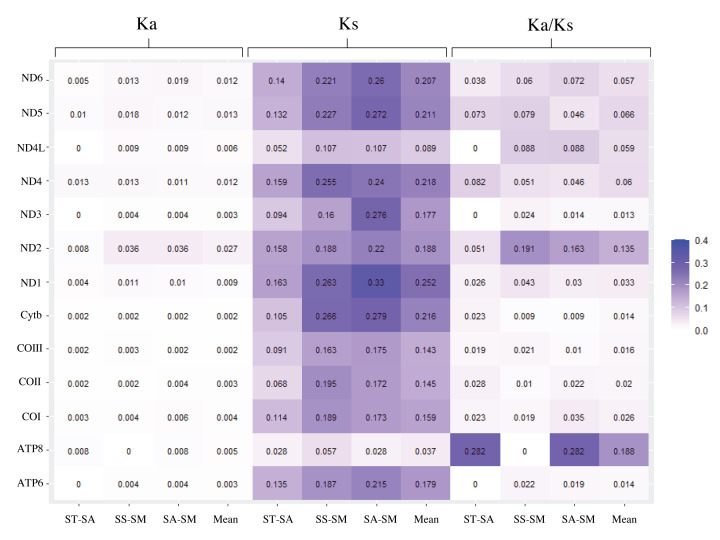
The rates of nonsynonymous substitutions and synonymous substitutions for each PCG in pairwise mitochondrial genome of *S. tertius* (ST), *S. albofasciatus* (SA), and *S. marmoratus* (SM)

The value of nonsynonymous substitution (*K*_a_)/synonymous substitution (*K*_s_) is a common indicator to assess selective pressure and evolutionary relationships of species in molecular studies [55]. *K*_a_/*K*_s_ < 1, *K*_a_/*K*_s_ = 1, and *K*_a_/*K*_s_ > 1 were represented purifying selection, neutral mutation, and positive selection, respectively [56]. All 13 PCG genes were under strong purifying selection with *K*_a_/*K*_s_ values below 1 (Figure 5). The result was different from deep-sea fishes, where most genes exhibited positive selection or convergent/parallel signals with the exception of ND4L and ND5 [57]. One of the reasons might be the different living environment between them. Environmental difference leads to the different expression levels of protein-coding, which involved in energy regulation, reproduction, and immune behaviors [26]. The basic characteristics of genome evolution depended on random genetic drift and mutation pressure that closely connected with the environment [58]. The deep-sea fishes inhabited in the condition of oxygen deficiency, lacked food, no sunlight, and extreme cold, while the *Sebastiscus* species survived in the warm coastal waters [57,59,60]. Positive selection usually related to the adaptation of new environments and the development of the new function, and most nonsynonymous mutations were disadvantage [61,62]. The *K*_a_/*K*_s_ values in *Sebastiscus* species showed they were under purifying selection, indicating that the environment variation was not great enough to change their genetic function.

ND2 and ATP8 genes had high *K*_a_/*K*_s_ (mean: 0.135, 0.188) values across three *Sebastiscus* mitogenomes compared with other genes, while ND3, ATP6, and Cytb genes were low (mean: 0.013, 0.014, 0.014). Low mutation rates tended to occur on highly expressed genes due to DNA repair mechanisms [63]. Compared with other genes, the ND3, ATP6, and Cytb showed low *K*_a_/*K*_s_ representing a low mutation rate, indicating that they may have higher expression level.

### rRNAs and tRNAs

Similar to *S. marmoratus*, the mitogenomes of *S. albofasciatus* and *S. tertius* each had one 12S rRNA and one 16SrRNA gene. The 12S rRNA gene was located between tRNA^Phe^ and tRNA^Val^, and the 16S rRNA gene was located between tRNA^Val^ and tRNA^Leu(UUR)^ as also occurs in some other Scorpaeniformes fishes [64,65]. The size of the 12S rRNA in *S. albofasciatus* and *S. marmoratus* were 946 bp, both a little shorter than in *S. tertius* (947 bp). The size of the 16S rRNA was 1692 bp in *S. albofasciatus* and *S. tertius*, which was consistent with *S. marmoratus*. In the *S. tertius* mitogenome, the A+T content of the rRNA genes was the minimum (52.25%) whereas the A+T content of rRNA in the *S. albofasciatus* and *S. marmoratus* mitogenomes were approximately 52.31 and 52.35%, lower than the control region. In three *Sebastiscus* species, the AT-skew of rRNA was strongly positive whereas the GC-skew was slightly negative indicating that the contents of A and C were higher than those of T and G in the rRNA, respectively.

**Figure 6:**
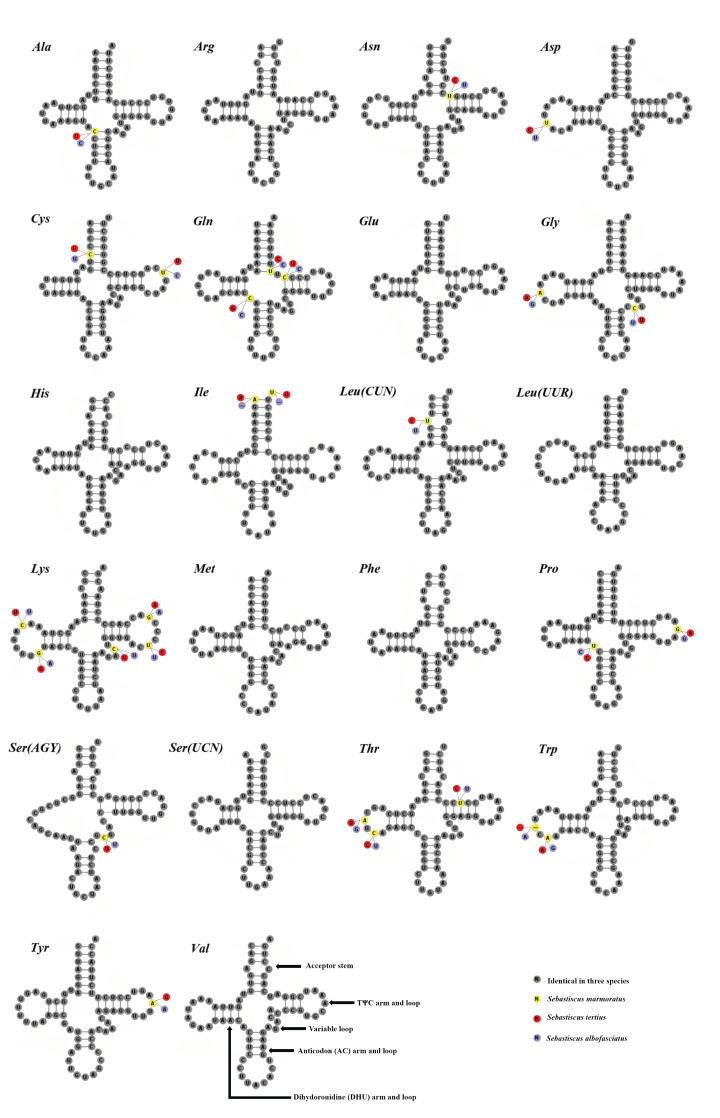
Inferred secondary structures of the 22 tRNA genes of *S. marmoratus, S. tertius*, and *S. albofasciatus* mitogenomes

Like the typical set of tRNA genes in Osteichthyes mitogenomes, there were 22 tRNA genes predicted in the three species. All of them had two kinds of tRNA^Leu^ and tRNA^Ser^ (Table 2). The secondary clover-leaf structures of tRNA genes identified in the mitogenome of *S. tertius, S. albofasciatus*, and *S. marmoratus* are shown in Figure 6. These tRNA genes varied in length from 65 to 74 bp. All the predicted tRNAs displayed the typical clover-leaf secondary structure, except for tRNA^Ser(AGY)^, which can not form a stable secondary structure because of lack of the DHU arm [66,67], this structure was common among fish mitogenomes [68].

### Control region

**Figure 7:**
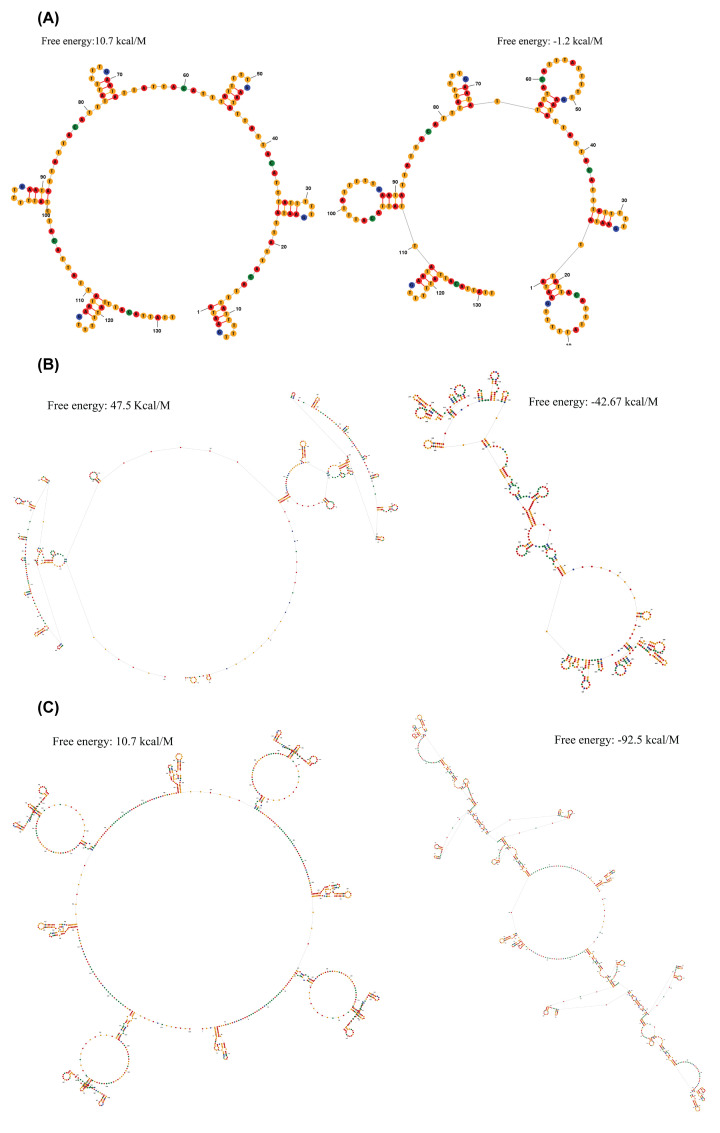
Predicted secondary structure and free energy of tandem repeat sequences about *S. tertius* (A), *S. albofasciatus* (B), and *S. marmoratus* (C) On the left is Maximum Expected Accuracy (MEA) and the right is Minimum Free Energy (MFE).

The control region is the largest non-coding region of the fish mitogenome which is equivalent to the A+T-rich region of the insect mitogenome [29,69]. The control regions of *Sebastiscus* mitogenomes were located between tRNA^Pro^ and tRNA^Phe^ with lengths of 1246 and 1391 bp, respectively (Table 2), much shorter than that of *S. marmoratus* (1918 bp). The length of the control region is variable, which is the main reason for the differences in mitochondrial DNA lengths in fishes [70–72]. In the mitogenomes of *S. tertius* and *S. albofasciatus*, the contents of A+T were 69.34 and 68.01%, respectively, similar to that of *S. marmoratus* (68.81%). The control regions of all three species genomes showed positive AT-skew values while all control regions of the three *Sebastiscus* species displayed negative GC-skew values. Additionally, we found tandem repetitive sequences in all three *Sebastiscus* species by Tandem Repeat Finder V 4.07 (http://tandem.bu.edu/trf/trf.html) [38]. The tandem repetitive sequence units of *S. tertius, S. albofasciatus* and *S. marmoratus* were 22, 275, and 269 bp, respectively. The secondary structures of tandem repetitive sequence for three species showed that the two kinds of structures can form multiple stem ring structures, respectively (Figure 7). As a non-coding region, the control region of mitochondrial DNA has a low evolutionary pressure. Therefore, it is frequency of base insertion and deletion is high, and the tandem repetitive sequence has often occurred, too. With the accumulation of data, the phenomenon of tandem repetition is becoming more and more frequent in the mitochondrial genome of fish [73]. The formation mechanism has been made some progress, of which slipped-strand mispairing is the most likely mechanism for tandem repeats [74,75]. In this study, the number of the core sequence repetitions in *S. tertius* and *S. albofasciatus* were 6 and 2, respectively, and that of *S. marmoratus* was 4. The difference between tandem repetitive sequence is the main reason the mitogenome lengths of the three species are different. Besides, the control region is responsible for the regulatory functions of DNA replication and transcription, which length variation will inevitably affect its functions and the metabolic frequency of the whole organism, thus resulting in interspecific differences.

### L-strand origin of replication

**Figure 8:**
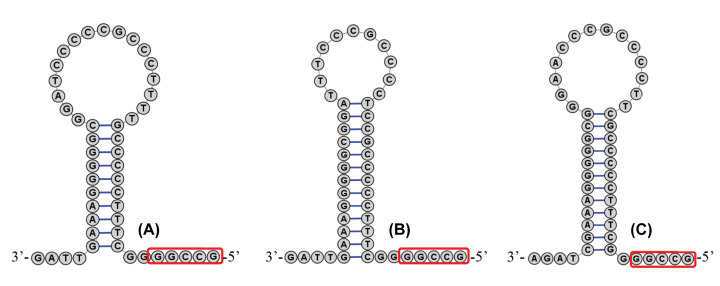
Inferred secondary structures of the OL of *Sebastiscus* mitogenome, *S. tertius* (A), *S. albofasciatus* (B), and *S. marmoratus* (C)

The L-strand origins of replication (O_L_) of *S. tertius* and *S. albofasciatus* mitogenomes were located between tRNA^Asn^ and tRNA^Cys^ with the same lengths of 37 bp, 1 bp shorter than *S. marmoratus* (38 bp). Secondary structures of O_L_ were displayed in Figure 8. In these O_L_ structures, the use of stem codons showed significant asymmetry, with more pyrimidines at the 5′ end of the sequence. Consistent of other studies in fish, all O_L_ regions had an identical conserved sequence region at the end of the stem (5′-GCCGG-3′) [45,76–78]. This may be related to the mechanism of RNA transformation to DNA [79]. Like found in many fishes, the use frequency of T and C bases in the ring region was higher [76,80].

### Phylogenetic analyses

**Figure 9:**
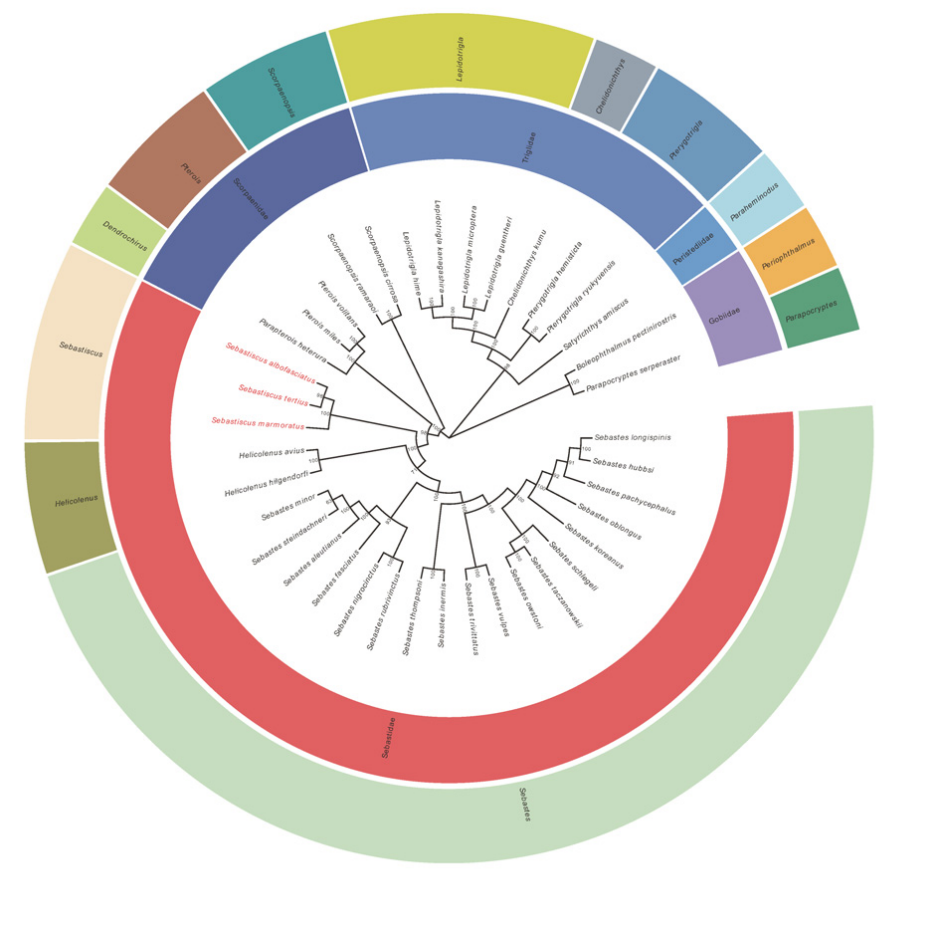
Phylogenetic tree of 36 Scorpaenoidei species constructed by BI and ML methods based on concatenated sequences of 13 PCGs *Boleophthalmus pectinirostris* and *Parapocryptes serperaster* were used as the outgroup. The species in red (Latin name) indicated the sequences generated in the present study. The numbers at codes showed the Bayesian posterior probabilities.

Phylogenetic relationships were reconstructed based on the sequences of 13 PCGs of 38 mitogenomes using BI and ML methods. The phylogenetic trees constructed by two methods were consistent with high intermediate bootstrap values, post probabilities, and the topological structure of the two phylogenetic trees were basically the same (Figure 9). We found three *Sebastiscus* species (*S. marmoratus, S. tertius*, and *S. albofasciatus*) formed a monophyletic group in both the BI and ML analyses. *S. tertius* and *S. albofasciatus* formed a sister group, which together had a sister relationship with *S. marmoratus*. Barsukov and Chen (1978) regarded *Sebastiscus* as a subgenus of *Sebastes* [17]. However, the result of our phylogenetic inference showed that *Helicolenus* are more closely related to *Sebastes* than to *Sebastiscus.* Our results supported the view that *Sebastiscus* should be treated as a genus independent from *Sebastes* which also was supported by some previous studies [11,81–83]. It was the strong evidence that supports the *Sebastiscus* as an independent species.

**Figure 10:**
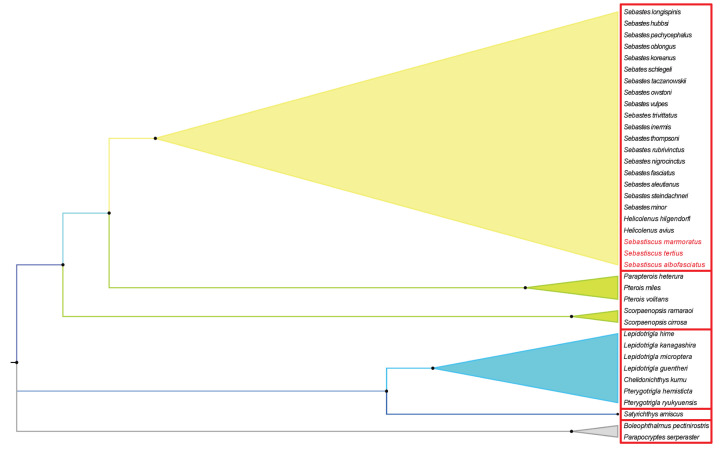
Phylogenetic relationships about Scorpaenoidei based on BI and ML methods by the concatenated sequences of 13 PCGs Each red frame represented a separate family.

Sebastidae and Scorpaenidae have been considered as two families by several authors [2–6]. Based on this study, however, if Sebastidae is valid, the family Scorpaenidae was regarded as paraphyletic with its subfamily Pteroinae in a sister relationship with Sebastidae (Figure 10). A number of authors thought that Sebastidae was considered as a subfamily of Scorpaenidae, and had an equal level with Pteroinae and Scorpaeninae [10,81,84]. Such taxonomic division was consistent with our results of phylogenetic relationships.

**Figure 11:**
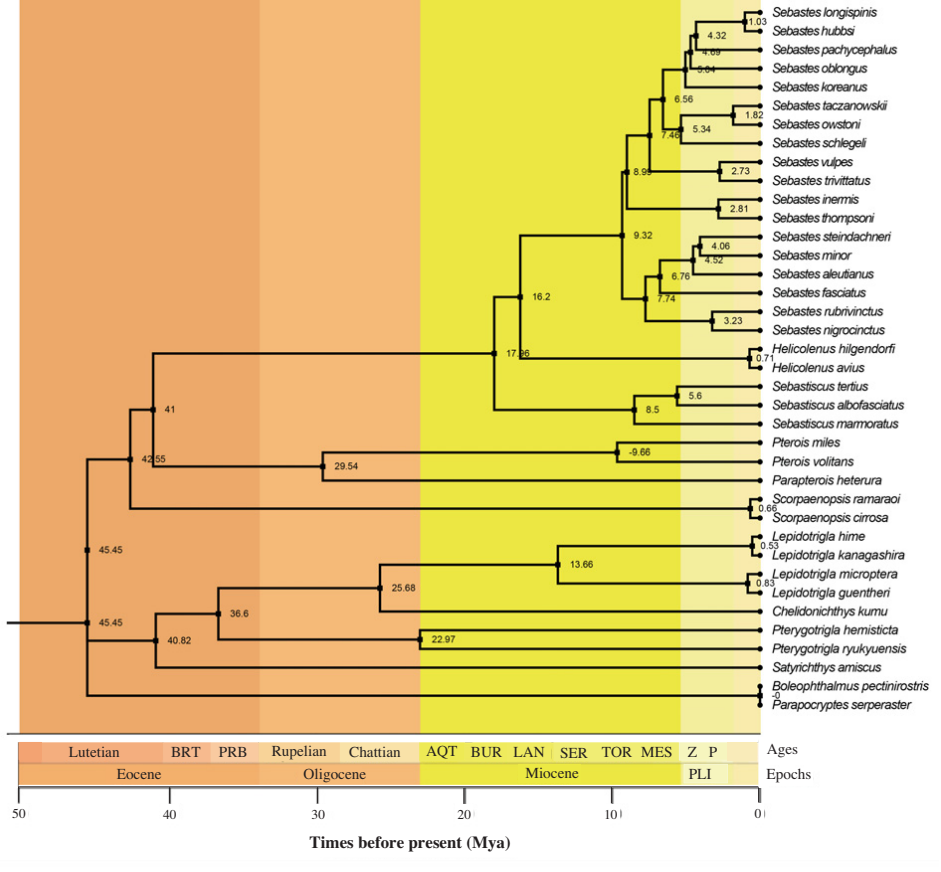
Divergence time analysis of 36 Scorpaenoidei fish species base on ML topology using concatenated sequences of 13 PCGs Numbers near the nodes indicated the estimated divergence time (Mya).

We estimated the species divergence time estimated using the ML method of RelTime by MEGA 10 (Figure 11). The divergence time calculations indicated that the Scorpaenidae had differentiated from other species ∼45.45 million years ago and all the families of Scorpaenoidei began to diverge more than 40 million years ago, except for Sebastidae. The differentiation time of Sebastidae from other species of Scorpaenidae was the lastest, which began ∼17.96 million years ago and the evolutionary rate of Sebastidae fishes were similar to the average rate of other subfamilies of Scorpaenidae. In summary, the taxonomic status of Sebastidae maybe coincides with the subfamilies of Scorpaenidae according to the result.

## Conclusions

In the present study, the complete mitogenomes of *S. tertius, S. albofasciatus*, and *S. marmoratus* were successfully determined. Their mitogenomes were with a total length of 16910 bp in *S. tertius*, 17056 bp in *S. albofasciatus*, and 17580 bp in *S. marmoratus*, respectively. A total of 22, 275, and 269 bp tandem repetitive sequences were respectively detected in three mitogenomes, which may be a typical characteristic of *Sebastiscus* fish. The ratio of *K*_a_ and *K*_s_ indicated that three species were suffering a purifying selection, while the ND2 and ATP8 showed the highest *K*_a_/*K*_s_ values. Both ML and BI analyses indicated that *Sebastiscus* was monophyletic and *S. marmoratus* was a sister clade to *S. tertius* and *S. albofasciatus*. That is to say, the taxonomic status of Sebastidae, well supported by results of a phylogenetic tree, should be subfamily Sebastinae. We believe the present study will greatly improve our understanding of the evolution profile and phylogenetic position in Scorpaeniformes, which could benefit resource management and species protection in fishery and aquaculture.

## Supplementary Material

Supplementary Figure S1 and Tables S1-S4

## Data Availability

The data used in the manuscript can be found on NCBI website (https://www.ncbi.nlm.nih.gov/) by accession number.

## Abbreviations

BI: Bayesian Inference
*K*_a_: nonsynonymous substitution
*K*_s_: synonymous substitution
ML: maximum likelihood
PCG: protein-coding gene
rRNA: ribosomal RNA
RSCU: relative synonymous codon usage
tRNA: transfer RNA

## References

[1] Nelson J.S. (2006) Fishes of the World, 4th edn, p. 468, John Wiley and Sons Press, New York, United States

[2] Ishida M. (1994) Phylogeny of the suborder Scorpaenoidei (Pisces: Scorpaeniformes). Bull. Nansei Natl. Fish. Res. Inst. 27, 1–112

[3] Imamura H. (2004) Phylogenetic relationships and new classification of the superfamily Scorpaenoidea (Actinopterygii: Perciformes). Species Diversity 9, 1–36 10.12782/specdiv.9.1

[4] Colton M.A. and Larson R.J. (2007) Aspects of the life history of treefish, *Sebastes serriceps* (Sebastidae). California Cooperative Oceanic Fisheries Investigations Report 48 10.1111/j.1365-2095.2007.00501.x

[5] Bakay Y.I. (2011) Ecologal and parasitological chracteristics of redfish *Sebastes mentella* (Scorpaeniformes: Sebastidae) from the Norwegian Sea and adjacent waters. J. Ichthyol. 51, 90–97 10.1134/S0032945211010036

[6] Morishita S., Kawai T. and Motomura H. (2018) *Sebastiscus vibrantus*, a new species of rockfish (Sebastidae) from Indonesia and Taiwan. Ichthyol. Res. 65, 423–432 10.1007/s10228-018-0632-9

[7] Eigenmann C.H. and Beeson C.H. (1894) A revision of the fishes of the subfamily Sebastinae of the Pacific coast of America. Proc. Natl. Museum 17, 375–407 10.5479/si.00963801.1009.375

[8] Barsukov V.V. (1981) A brief review of the subfamily Sebastinae. J. Ichthyol. 21, 1–26

[9] Nelson J.S. (1994) Fishes of the World, 3rd edn, p. 600, John Wiley and Sons Press, New York, United States

[10] Katoh M. and Tokimura M. (2001) Genetic and morphological identification of *Sebastiscus tertius* in the east china sea (Scorpaeniformes: Scorpaenidae). Ichthyol. Res. 48, 247–255

[11] Xu T.J., Cheng Y.Z., Liu X.Z. et al. (2011) The complete mitochondrial genome of the marbled rockfish *Sebastiscus marmoratus* (Scorpaeniformes, Scorpaenidae): genome characterization and phylogenetic considerations. Mol. Biol. 45, 434–445 10.1134/S0026893311020191 21790005

[12] Jang Y.S., Park K.J., Kim K.Y. et al. (2016) The complete mitochondrial genome of *Sebastes pachycephalus* (Scorpaenidae, Scorpaeniformes) from the east sea, Korea. Mitochondrial DNA Part A 27, 69–70 10.3109/19401736.2013.873904 24438257

[13] Jordan D.S. and Starks E.C. (1904) A review of the Japanese fishes of the family of Agonidae. Proc. U.S. Natl. Museum 27, 575–599 10.5479/si.00963801.27-1365.575

[14] Chen D.G. and Zhang M.Z. (2015) Marine Fishes of China, 1st edn, pp. 764–765, Ocean University of China Press, Qingdao, China

[15] Matsubara K. (1943a) Studies on the Scorpaenoid fishes of Japan. Anatomy, phylogenya and taxonomy (I). Trans Shigenkagaku Kenkyusyo 1, 1–170

[16] Matsubara K. (1943b) Studies on the Scorpaenoid fishes of Japan. Anatomy, phylogenya and taxonomy (II). Trans. Shigenkagaku Kenkyusyo 2, 171–485

[17] Barsukov V.V. and Chen L.C. (1978) Review of the subgenus *Sebastiscus* (Sebastes, Scorpaenidae) with a description of a new species. Voprosy Ikhtiol. 18, 195–210

[18] Washington B.B., Eschmeyer W.N. and Howe K.M. (1984) Scorpaeniformes: relationships. In Ontogeny and Systematics of Fishes(Moser H.G., Richards W.J., Cohen D.M., Fahay M.P. and Kendall A.W. Jr Richar dson S.L., eds), pp. 438–447, American Society of Ichthyologists and Herpetologists, New York, United States

[19] Amaoka K. (1984) Sebastiscus tertius. In The Fishes of The Japanese Archipelago(Masuda H. and Amaoka K., eds), p. 313, Tokai University Press, Tokyo, Japan

[20] Shimizu T. (1984a) *Sebastiscus marmoratus* (Cuvier). In The Fishes of The Japanese Archipelago(Masuda H., Amaoka K., Araga C., Uyeno T. and Yoshino T., eds), p. 313, Tokai University Press, Tokyo, Japan

[21] Shimizu T. (1984b) *Sebastiscus albofasciatus* (Lacepède). In The Fishes of The Japanese Archipelago(Masuda H., Amaoka K., Araga C., Uyeno T. and Yoshino T., eds), p. 313, Tokai University Press, Tokyo, Japan

[22] Lacepède H. (1802) Histoire Naturelle des Poissons., vol. 4, Plassan, Paris, France

[23] Cuvier G. and Valenciennes A. (1829) Histoire naturelle des poissons. Livre quatrième. Des acanthoptérygiens à joue cuirassée., vol. 4, Levrault, Paris, France

[24] Nakabo T. and Kai Y. (2013) Sebastidae. In Fishes of Japan with Pictorial Keys to The Species. 3rd edn, (Nakabo T., ed.), pp. 1933–1938, Tokai University Press, Hadano, Japan

[25] Groves P. and Shields G.F. (1996) Phylogenetics of the caprinae based on cytochrome b sequence. Mol. Phylogenet. Evol. 5, 467–476 10.1006/mpev.1996.0043 8744761

[26] Yan C., Duanmu X., Zeng L. et al. (2019) Mitochondrial DNA: distribution, mutations, and elimination. Cells 8, 379 10.3390/cells8040379 PMC6523345 31027297

[27] Nielsen J.L., Graziano S.L. and Seitz A.C. (2010) Fine-scale population genetic structure in Alaskan Pacific halibut (*Hippoglossus stenolepis*). Conserv. Genet. 11, 999–1012 10.1007/s10592-009-9943-8

[28] Cheng J., Ma G.Q., Miao Z.Q. et al. (2012) Complete mitochondrial genome sequence of the spiny head croaker *Collichthys lucidus* (Perciformes, Sciaenidae) with phylogenetic considerations. Mol. Biol. Rep. 39, 4249–4259 10.1007/s11033-011-1211-6 21786157

[29] Zhang L., Cai Y.Y., Yu D.N. et al. (2018) Gene characteristics of the complete mitochondrial genomes of *Paratoxodera polyacantha* and *Toxodera hauseri* (Mantodea: Toxoderidae). PeerJ 6, e4595 10.7717/peerj.4595 29686943 PMC5911385

[30] Bresch H.F. (1984) Hybridization and introgression among species of sunfish (*Lepomis*): analysis by mitochondrial DNA and allozyme markers. Genetics 108, 237–255 6090268 10.1093/genetics/108.1.237 PMC1202397

[31] Michaclis G.S. (1982) Mitochondrial DNA copy number in a bovine ocytes and somatic cell. Dev. Biol. 97, 246–251 10.1016/0012-1606(82)90088-4 6295849

[32] Yang H., Xia J., Zhang J.E. et al. (2018) Characterization of the complete mitochondrial genome sequences of three croakers (Perciformes, Sciaenidae) and novel insights into the phylogenetics. Int. J. Mol. Sci. 19, 17–41 10.3390/ijms19061741 PMC6032254 29895774

[33] Johansen S., Guddal P.H. and Johansen T. (1990) Organization of the mitochondrial genome of Atlantic cod, *Gadus morhua*. Nucleic Acids Res. 18, 411–419 10.1093/nar/18.3.411 2308841 PMC333442

[34] Stepien C.A. (1997) Molecular systematics of fishes. In Molecules and Morphology in Studies of Fish Evolution(Carol A.S. and Thomas D.K., eds), pp. 1–11, Academic Press, Salt Lake, United States

[35] Miya M. and Nishida M. (2000) Use of mitogenomic information in teleostean molecular phylogenetics: A tree-based exploration under the maximum parsimony optimality criterion. Mol. Phylogenet. Evol. 17, 437–455 10.1006/mpev.2000.0839 11133198

[36] Sing V.K., Mangalam A.K., Dwivedi S. et al. (1998) Primer premier: program for design of degenerate primers from a protein sequence. BioTechniques 24, 318–319 10.2144/98242pf02 9494736

[37] Lowe T.M. and Chan P.P. (2016) tRNAscan-SE on-line: search and contextual analysis of transfer RNA genes. Nucleic Acids Res. 44, 54–57 10.1093/nar/gkw413 PMC4987944 27174935

[38] Benson G. (1999) Tandem repeats finder: a program to analyze DNA sequences. Nucleic Acids Res. 27, 573–580 10.1093/nar/27.2.573 9862982 PMC148217

[39] Iwasaki W., Fukunaga T., Isagozawa R. et al. (2013) MitoFish and MitoAnnotator: a mitochondrial genome database of fish with an accurate and automatic annotation pipeline. Mol. Biol. Evol. 30, 2531–2540 10.1093/molbev/mst141 23955518 PMC3808866

[40] Sato Y., Miya M., Fukunaga T. et al. (2018) MitoFish and MiFish Pipeline: a mitochondrial genome database of fish with an analysis pipeline for environmental DNA metabarcoding. Mol. Biol. Evol. 35, 1553–1555 10.1093/molbev/msy074 29668970 PMC5967551

[41] Tamura K., Peterson D., Peterson N. et al. (2011) MEGA5: Molecular evolutionary genetics analysis using maximum likelihood, evolutionary distance, and maximum parsimony methods. Mol. Biol. Evol. 28, 2731–2739 10.1093/molbev/msr121 21546353 PMC3203626

[42] Ronquist F., Teslenko M., Mark P. et al. (2012) MrBayes 3.2: Ecient Bayesian Phylogenetic Inference and model choice across a large model space. System. Biol. 61, 539–542 10.1093/sysbio/sys029 22357727 PMC3329765

[43] Sudhir K., Glen S., Michael L. et al. (2018) MEGA X: molecular evolutionary genetics analysis across computing platforms. Mol. Biol. Evol. 6 10.1093/molbev/msy096 PMC5967553 29722887

[44] Kumar S., Stecher G., Suleski M. et al. (2017) TimeTree: a resource for timelines, timetrees, and divergence times. Mol. Biol. Evol. 34, 1812–1819 10.1093/molbev/msx116 28387841

[45] Oh D.J., Kim J.Y., Lee J.A. et al. (2007) Complete mitochondrial genome of the rock bream *Oplegnathus fasciatus* (Perciformes, Oplegnathidae) with phylogenetic considerations. Gene 392, 174–180 10.1016/j.gene.2006.12.007 17258872

[46] Catanese G., Manchado M. and Infante C. (2010) Evolutionary relatedness of mackerels of the genus *Scomber* based on complete mitochondrial genomes: strong support to the recognition of Atlantic *Scomber colias* and Pacific *Scomber japonicus* as distinct species. Gene 452, 35–43 10.1016/j.gene.2009.12.004 20035845

[47] Li Y., Song N., Lin L. et al. (2014) The complete mitochondrial genome of *Pampus echinogaster* (Perciformes: Stromateidae). Mitochondrial DNA Part A 27, 289–290 10.3109/19401736.2014.892081 24617460

[48] Li N., Song N. and Gao T.X. (2015) The complete mitochondrial genome of Japanese *Ammodytes personatus* (Perciformes, Ammodytidae). Mitochondrial DNA 26, 781–782 10.3109/19401736.2013.855750 24409904

[49] Xiao J., Song N., Gao T. and Roland J.M. (2014a) The complete mitochondrial genome of *Sillago indica* (Perciformes: Sillaginidae). Mitochondrial DNA Part A 27, 1445–1446 10.3109/19401736.2014.953085 25231721

[50] Xiao J., Song N., Gao T. and Zhao Y. (2014b) The complete mitochondrial genome of *Sillago asiatica* (Perciformes: Sillaginidae). Mitochondrial DNA Part A 27, 1644–1645 10.3109/19401736.2014.958709 25231722

[51] Liu Z., Song N., Yanagimoto T. et al. (2017) Complete mitochondrial genome of three fish species (Perciformes: Amblyopinae): Genome description and phylogenetic relationships. Pakistan J. Zool. 49, 107–115 10.17582/journal.pjz/2017.49.1.107.115

[52] Ruan H., Li M., Li Z. et al. (2020) Comparative analysis of complete mitochondrial genomes of three *Gerres* fishes (Perciformes: Gerreidae) and primary exploration of their evolution history. Int. J. Mol. Sci. 21, 1874 10.3390/ijms21051874 PMC7084342 32182936

[53] Ojala D., Montoya J. and Attardi G. (1981) tRNA punctuation model of RNA procession in human mitochondria. Nature 290, 470–474 10.1038/290470a0 7219536

[54] Nei M. (1972) Genetic distance between populations. Am. Nat. 106, 283–292 10.1086/282771

[55] Zhu K.C., Liang Y.Y., Wu N. et al. (2017) Sequencing and characterization of the complete mitochondrial genome of Japanese Swellshark (*Cephalloscyllium umbratile*). Sci. Rep. 7, 15299 10.1038/s41598-017-15702-0 29127415 PMC5681689

[56] Yang Z. and Bielawski J.P. (2000) Statistical methods for detecting molecular adaptation. Trends Ecol. Evol. 15, 496–503 10.1016/S0169-5347(00)01994-7 11114436 PMC7134603

[57] Shen X., Pu Z., Chen X. et al. (2019) Convergent evolution of mitochondrial genes in deep-sea fishes. Front. Genet. 10, 925 10.3389/fgene.2019.00925 31632444 PMC6785628

[58] Schaack S., Ho E.K. and Macrae F. (2019) Disentangling the intertwined roles of mutation, selection and drift in the mitochondrial genome. Philos. Trans. R. Soc. B 375, 20190173 10.1098/rstb.2019.0173 PMC6939366 31787045

[59] Teixeira R.L. and Helmer J.L. (1997) Ecology of young mojarras (Pisces: Gerreidae) occupying the shallow waters of a tropical estuary. Rev. Bras. Biol. 57, 637–646

[60] Chollet V.J., DeLa C.A.J. and Garcia R.F.J. (2014) Comparison of urohyal bone morphology among gerreid fish (Perciformes: Gerreidae). Italian J. Zool. 81, 246–255 10.1080/11250003.2014.912681

[61] Nielsen R. (2005) Molecular signatures of natural selection. Annu. Rev. Genet. 39, 197–218 10.1146/annurev.genet.39.073003.112420 16285858

[62] Xiang D., Shen X., Pu Z. et al. (2018) Convergent evolution of human-isolated H7N9 avian influenza a viruses. J. Infect. Dis. 217, 1699–1707 10.1093/infdis/jiy082 29438519

[63] Barrientos A., Barros M.H., Valnot I. et al. (2002) Cytochrome oxidase in health and disease. Gene 286, 53–63 10.1016/S0378-1119(01)00803-4 11943460

[64] Song Y.S., Kim I.H., Kim Y.K. et al. (2016) Complete mitochondrial genome of *Hemilepidotus gilberti*, (Scorpaeniformes: Cottidae). Mitochondrial DNA Part B 1, 962–963 10.1080/23802359.2016.1266706 PMC7800366 33490430

[65] Dray L., Neuhof M., Diamant A. et al. (2016) The complete mitochondrial genome of the devil firefish *Pterois miles* (Bennett, 1828) (Scorpaenidae). Mitochondrial DNA 27, 783–784 10.3109/19401736.2014.945565 25103446

[66] Anderson S., Bankier A.T., Barrell B.G. et al. (1981) Sequence and organization of the human mitochondrial genome. Nature 290, 457–465 10.1038/290457a0 7219534

[67] Frazer-Abel A.A. and Hagerman P.J. (1999) Determination of the angle between the acceptor and anti codon stems of a truncated mitochondrial tRNA. J. Mol. Biol. 285, 581–593 10.1006/jmbi.1998.2320 9878431

[68] Manehado M., Catanese G. and Infantete C. (2004) Complete mitochondrial DNA sequence of the Ailantic Bluefin tuna *Thunnus thynnus*. Fisheries Sci. 70, 68–70 10.1111/j.1444-2906.2003.00772.x

[69] Du C., He S.L., Song X.H. et al. (2016) The complete mitochondrial genome of *Epicauta chinensis* (Coleoptera: Meloidae) and phylogenetic analysis among coleopteran insects. Gene 578, 274–280 10.1016/j.gene.2015.12.036 26707213

[70] Jin X., Liu X. and Sun Y. (2013) Complete mitochondrial genome of the greenspot goby *Acentrogobius chlorostigmatoides* (Perciformes, Gobioidei): repetitive sequences in the control region. Mitochondrial DNA 24, 400–402 10.3109/19401736.2013.763244 23516980

[71] Jin X., Wang R., Wei T. et al. (2015) Complete mitochondrial genome sequence of *Tridentiger bifasciatus* and *Tridentiger barbatus* (Perciformes, Gobiidae): a mitogenomic perspective on the phylogenetic relationships of Gobiidae. Mol. Biol. Rep. 42, 253–265 10.1007/s11033-014-3768-3 25260906

[72] Xiao J. (2015) The Complete Mitochondrial Genomes and Phylogenetic Analysis of *Sillago* species. Ocean University of China, Qingdao, Shandong, China, Masters Thesis

[73] Vodolazhskii D.I., Kornienko I.V. and Voinova N.V. (2008) Hypervariability of the D-loop region in mitochondrial DNA of Russian sturgeon *Acipenser gueldenstaedtii* (Acipenseriformes, Acipenseridae). J. Ichthyol. 48, 188–197 10.1134/S0032945208020057

[74] Rand D.M. and Harrison R.G. (1989) Molecular population genetics of mtDNA size variation in crickets. Genetics 121, 551–569 2565855 10.1093/genetics/121.3.551 PMC1203640

[75] Wilkinson G.S. and Chapman A.M. (1991) Length and sequence variation in evening bat D-loop mtDNA. Genetics 128, 607–617 1874418 10.1093/genetics/128.3.607 PMC1204534

[76] Zardoya R., Garrido P.A. and Bautista J.M. (1995) The complete nucleotide sequence of the mitochondrial DNA genome of the rainbow trout, *Oncorhynchus mykiss*. J. Mol. Evol. 41, 942–951 10.1007/BF00173174 8587139

[77] Noack K., Zardoya R. and Meyer A. (1996) The complete mitochondrial DNA sequence of the bichir (*Polypterus ornatipinnis*), a basal ray-finned fish: ancient establishment of the consensus vertebrate gene order. Genetics 144, 1165–1180 8913758 10.1093/genetics/144.3.1165 PMC1207609

[78] Fonseca M.M. and Harris D.J. (2008) Relationship between mitochondrial gene rearrangements and stability of the origin of light strand replication. Genet. Mol. Biol. 31, 566–574 10.1590/S1415-47572008000300027

[79] Hixson J.E., Wong T.W. and Clayton D.A. (1986) Both the conserved stem-loop and divergent 5′-flanking sequences are required for initiation at the human mitochondrial origin of light-strand DNA replication. J. Biol. Chem. 261, 2384–2390 3944140

[80] Cheng Y.Z., Xu T.J., Shi G. et al. (2010) Complete mitochondrial genome of the miiuy croaker *Miichthys miiuy* (Perciformes, Sciaenidae) with phylogenetic consideration. Mar. Genomics 3, 201–209 10.1016/j.margen.2010.10.003 21798214

[81] Ishii T. (1997) The study of speciation from analysis of mitochondrial DNA structures among the fishes in Scorpaenidae. Sophia Life 16, 105–111

[82] Yoshiaki K., Nakayama K. and Nakabo T. (2003) Molecular phylogenetic perspective on speciation in the genus *Sebastes* (Scorpaenidae) from the Northwest Pacific and the position of Sebastes within the subfamily Sebastinae. Ichthyol. Res. 50, 239–244

[83] John R.H. and Rusell D.V. (2007) The origin, evolution and diversification of rockfishes of the genus *Sebastes* (Cuvier). Mol. Phylogenet. Evol. 44, 790–811 17320419 10.1016/j.ympev.2006.12.026

[84] Nelson J.S., Grande T.C. and Wilson M.V.H. (2016) Fishes of the World, 5th edn, p. 321, John Wiley and Sons Press, New York, United States

