# Supplementary material for: Comparative analysis of the complete mitochondrial genomes of three rockfishes (Scorpaeniformes, Sebastiscus) and insights into the phylogenetic relationships of Sebastidae
Source: Biosci Rep. 2020 Dec 14;40(12):BSR20203379. doi: 10.1042/BSR20203379 (PMC7736627; doi:10.1042/BSR20203379)
Supplement: Supplementary Figure S1 and Tables S1-S4 [file BSR-2020-3379_supp.pdf]

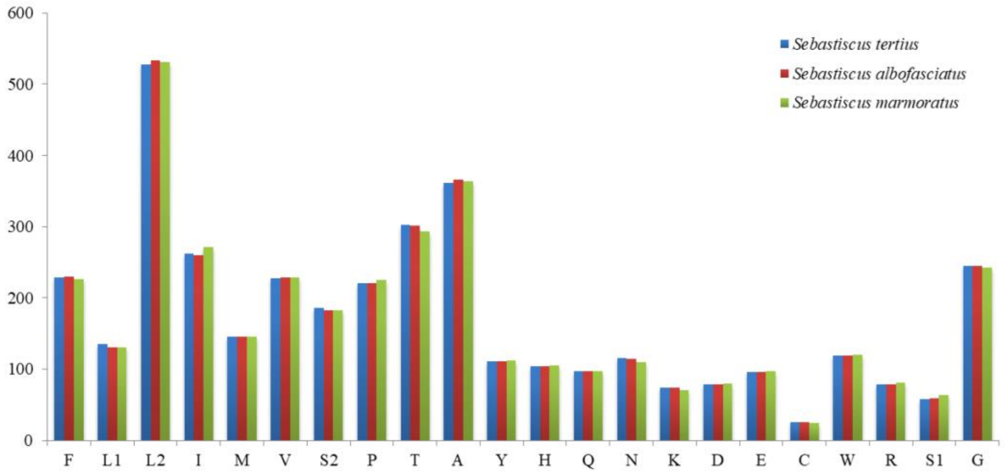

600
500
400
300
200
100
0
F
L1
L2
I
M
V
S2
P
T
A
Y
H
Q
N
K
D
E
C
W
R
S1
G
Sebastiscus tertius
Sebastiscus albofasciatus
Sebastiscus marmoratus

Supplementary Figure S1. Total codons in *S. tertius, S. albofasciatus,* and *S. marmoratus*.

Table S1 *Sebastiscus*-specific universal primer sets.

Primer name	Sequence (5'–3')	Product Length (bp)	Primer name	Sequence (5'–3')	Product length (bp)
S-1-F	AAGGCTTGGTCCTGACTT	651	S-18-F	AACCTGACCATGACACTAAG	632
S-1-R	TTGTGCCAGTTGTGCTTA		S-18-R	GCTACAGCAACTTCTAATAGG	
S-2-F	TATACGCATCCGAAGGTTAG	655	S-19-F	CTGGTCACCTCTTAATTCAAC	653
S-2-R	GGTGTCTCTTCAGTGTAAGG		S-19-R	TTCGCTCTCCTTCCATAATG	
S-3-F	GGAGGCAAGTCGTAACAT	543	S-20-F	GACCTCCTACGGGCATTA	563
S-3-R	GGCTGAACTTCTATCCAATTC		S-20-R	GGATTACGGAGAGCACAATA	
S-4-F	AACCTACCGAACCTAGTTATAG	541	S-21-F	CTACTGATGAGGCTCTTAATCT	580
S-4-R	TGCCGAGTTCCTTCTCTT		S-21-R	GGTTCGATGGAATGCTAGG	
S-5-F	GGAGAGACCCTGCTAATATG	629	S-22-F	GTCCATAATTGTCTGATGACTC	573
S-5-R	GCGGCTCTTAGTTGTAGG		S-22-R	TAGGCTCAAGTGCTGTGT	
S-6-F	GCCTGAACTAATGGAACC	551	S-23-F	ATCCTGCCTTACACCATTC	651
S-6-R	TAAGGAGAGGACTTGAACC		S-23-R	CAGGTTGAGCCAGTCATAA	
S-7-F	AATAGGCAAGAGGGCATAC	556	S-24-F	ACTAGGAGGATACGGTATAATC	615
S-7-R	GAAATGGTTTGGGCTACG		S-24-R	AGGAGGTGGAGGGCTATA	
S-8-F	ACTCACACTTGCCTTAACC	585	S-25-F	ATGAACCCTTGTCCTAACG	634
S-8-R	ACTCACAGGAATAGGACAGA		S-25-R	ATGAGGTGATGATTGCTTCT	
S-9-F	TAGCCGAATATGCCAACAT	566	S-26-F	ACCCAATCTTTACAACCCTA	666
S-9-R	CTTGCGAAGGTAGTAGTAGT		S-26-R	TAGCAGAGGGAAGTCAGG	
S-10-F	TCTTAACCGCCCTCCTATT	557	S-27-F	TAGCGTGAATAGCAACCAA	622
S-10-R	TACTATTCAGCCGAGATGTG		S-27-R	TTGGAGAAGAATCCTGCTAG	
S-11-F	ATCCTTGGACTAACCTCAAC	661	S-28-F	ACAGCCTTAATGACGAACA	668
S-11-R	GTCCCGCAAGTCTTATCA		S-28-R	TGATGAAGAAATGGCTTTGG	
S-12-F	CTACGCTATTGCTCCTACC	631	S-29-F	CACACCACTTCTCCAATATAC	599
S-12-R	GCATGTGCTGTAACGATTA		S-29-R	TGGGATGTTAGTTGTGTTTG	
S-13-F	CGAGCAGAATTAAGCCAAC	641	S-30-F	CACAACAGGACCACTCAC	591
S-13-R	AAGCCAGGGAGGATAAGAA		S-30-R	GGCGTGTAGATTTCGGATA	
S-14-F	GGTATCACGATGCTTCTAAC	644	S-31-F	TAGCCATACATTACACCTCT	606
S-14-R	AAGAACATAATGCCGAAGTG		S-31-R	GGTCTCCTAGCAGATTGG	
S-15-F	CCACTTCCACTATGTTCTGT	533	S-32-F	ACCCACTTGGTCTAAACTC	607
S-15-R	GGCGGTGTAATGTGTTCT		S-32-R	TCTCCTTTCTTTGAGCAGTA	
S-16-F	TCAAGCCGACCACATAAC	602	S-33-F	TAGTAGCTCAGCGTCAGA	642
S-16-R	GAGTGGAGTACATCGTCTG		S-33-R	CCTTGCCCTATGGAGAGA	
S-17-F	GACAATTCCGCCTATTAGAAG	627	S-34-F	TAGTAAGAACCGACCAACAA	479
S-17-R	GGTAGAGGAGTCAAGGTAAG		S-34-R	GGCTTAGTTATCTCAGGAGTT	

Table S2. Specific primers used to amplify the mitogenomes of *S. tertius* and *S. albofasciatus*.

Species	Primer name	Sequence (5'–3')	Product length
Sebastiscus tertius	S-36-F	GTTCAACCTACACTGGCATA	665
	S-36-R	GTGGTTTAGCTTCTCACTTATG	
	S-37-F	AACTCCTGAGATAACTAACG	602
	S-37-R	CGGATACTTGCATGTGTAA	
Sebastiscus albofasciatus	S-48-F	GGGTTATCAAGGACATAAGTAG	850
	S-48-R	GCTGTAGGGCATTCTCAC	
Sebastiscus marmoratus	S-49-F	TCAAGGACATAAGTGAGGAT	1237
	S-49-R	CAGGACCAAGCCTTTGTG	

Table S3 Base composition of *Sebastiscus* mitochondrial genomes.

	S. tertius					***S. albofasciatus***					***S. marmoratus***				
	**A**	**T**	**G**	**C**	**A+T**	**A**	**T**	**G**	**C**	**A+T**	**A**	**T**	**G**	**C**	**A+T**
Whole	28.30	26.70	16.60	28.40	55.04	28.44	26.71	16.65	28.20	55.15	28.87	26.75	16.31	28.07	55.65
PCGs-H	26.10	28.39	15.33	30.19	54.48	26.17	28.51	15.30	30.02	54.68	26.08	28.42	15.39	30.11	54.50
PCGs-L	14.37	37.55	34.10	13.98	51.92	14.40	36.80	33.70	15.10	51.20	14.75	38.51	33.33	13.41	53.26
rRNAs	31.49	20.77	21.67	26.07	52.26	31.61	20.70	21.57	26.12	52.31	31.77	20.58	21.46	26.19	52.35
D-loop	35.15	34.19	12.76	17.90	69.34	35.87	32.14	13.08	18.91	68.01	37.02	31.25	12.58	19.15	68.81
ATP6	26.94	29.87	13.62	29.58	56.81	26.79	29.87	13.47	29.87	56.66	27.23	28.99	13.03	30.75	56.22
ATP8	29.17	23.21	13.10	34.52	52.38	28.57	22.62	13.69	35.12	51.19	29.17	22.02	13.10	35.71	51.19
COI	24.56	30.37	18.18	26.89	54.93	24.95	30.37	17.86	26.82	55.32	24.80	30.60	17.70	26.80	55.40
COII	28.80	28.08	16.50	26.63	56.87	29.09	28.22	16.21	26.48	57.31	28.20	28.70	16.60	26.50	56.90
COIII	25.61	27.77	17.07	29.55	53.38	25.10	27.39	17.32	30.19	52.48	25.50	27.60	16.90	29.90	53.10
Cytb	25.33	30.32	15.25	29.10	55.65	25.24	30.15	15.07	29.54	55.39	24.80	30.85	15.43	28.92	55.65
ND1	24.82	27.28	15.18	32.72	52.10	24.72	27.90	15.59	31.79	52.62	24.92	27.49	15.49	32.10	52.41
ND2	27.63	26.48	12.24	33.65	54.11	27.34	26.29	12.52	33.84	53.63	26.80	26.70	13.10	33.40	53.60
ND3	21.78	32.38	15.76	30.09	54.15	21.78	32.66	16.05	29.51	54.44	21.78	32.95	16.05	29.23	54.73
ND4	26.00	28.31	15.28	30.41	54.31	26.29	28.60	14.99	30.12	54.89	26.34	27.65	14.88	31.13	53.99
ND4L	20.88	28.28	17.17	33.67	49.16	21.89	28.62	16.50	33.00	50.51	22.22	27.61	16.50	33.67	49.83
ND5	28.00	26.81	14.14	31.05	54.81	28.22	27.24	14.25	30.29	55.46	28.11	26.97	14.68	30.23	55.08
ND6	14.37	37.55	34.10	13.98	51.92	14.37	36.78	33.72	15.13	51.15	14.75	38.51	33.33	13.41	53.26

Table S4 The codon number and relative synonymous codon usage in mitochondrial protein coding genes.

Condon	Count			RSCU			Condon	Count			RSCU			Condon	Count			RSCU		
	ST	SA	SM	ST	SA	SM		ST	SA	SM	ST	SA	SM		ST	SA	SM	ST	SA	SM
UUU(F)	110	112	111	0.96	0.97	0.98	UCU(S)	41	40	38	1.01	1	0.93	UAU(Y)	48	54	60	0.86	0.97	1.08
UUC(F)	119	118	116	1.04	1.03	1.02	UCC(S)	64	62	69	1.57	1.54	1.68	UAC(Y)	63	57	51	1.14	1.03	0.92
UUA(L)	108	104	100	0.98	0.94	0.91	UCA(S)	77	75	73	1.89	1.87	1.78	AGU(S)	19	18	18	0.47	0.45	0.44
UUG(L)	27	26	30	0.24	0.24	0.27	UCG(S)	4	5	4	0.1	0.12	0.1	AGC(S)	39	41	44	0.96	1.02	1.07
CUU(L)	154	162	157	1.39	1.47	1.43	CCU(P)	57	55	46	1.04	1	0.84	CAU(H)	32	30	32	0.62	0.58	0.6
CUC(L)	142	130	136	1.29	1.18	1.23	CCC(P)	93	98	106	1.69	1.78	1.94	CAC(H)	72	74	74	1.38	1.42	1.4
CUA(L)	186	200	181	1.68	1.81	1.64	CCA(P)	57	56	59	1.04	1.02	1.08	CAA(Q)	87	82	83	1.79	1.69	1.71
CUG(L)	46	41	57	0.42	0.37	0.52	CCG(P)	13	11	8	0.24	0.2	0.15	CAG(Q)	10	15	14	0.21	0.31	0.29
AUU(I)	162	164	173	1.24	1.26	1.29	ACU(T)	58	56	54	0.77	0.74	0.72	AAU(N)	54	51	53	0.94	0.89	0.94
AUC(I)	100	96	96	0.76	0.74	0.71	ACC(T)	111	111	113	1.47	1.48	1.51	AAC(N)	61	63	60	1.06	1.11	1.06
AUA(M)	90	94	90	1.24	1.29	1.23	ACA(T)	116	117	120	1.53	1.55	1.61	AAA(K)	64	64	68	1.73	1.73	1.86
AUG(M)	55	52	56	0.76	0.71	0.77	ACG(T)	18	17	12	0.24	0.23	0.16	AAG(K)	10	10	5	0.27	0.27	0.14
GUU(V)	61	72	64	1.07	1.26	1.13	GCU(A)	76	73	73	0.84	0.8	0.8	GAU(D)	23	26	28	0.58	0.66	0.71
GUC(V)	58	50	56	1.02	0.87	0.99	GCC(A)	160	168	164	1.77	1.84	1.81	GAC(D)	56	53	51	1.42	1.34	1.29
GUA(V)	81	82	83	1.42	1.43	1.46	GCA(A)	107	110	110	1.19	1.2	1.21	GAA(E)	73	75	77	1.52	1.56	1.6
GUG(V)	28	25	24	0.49	0.44	0.42	GCG(A)	18	15	16	0.2	0.16	0.18	GAG(E)	23	21	19	0.48	0.44	0.4
UGU(C)	6	6	5	0.46	0.46	0.38	CGU(R)	8	8	8	0.41	0.41	0.41	GGU(G)	37	35	43	0.6	0.57	0.7
UGC(C)	20	20	21	1.54	1.54	1.62	CGC(R)	19	18	18	0.96	0.91	0.92	GGC(G)	90	92	82	1.47	1.5	1.34
UGA(W)	106	106	104	1.78	1.78	1.75	CGA(R)	44	39	42	2.23	1.97	2.15	GGA(G)	79	82	85	1.29	1.34	1.39
UGG(W)	13	13	15	0.22	0.22	0.25	CGG(R)	8	14	10	0.41	0.71	0.51	GGG(G)	39	36	35	0.64	0.59	0.57

Notes: ST, *S. tertius*; SA, *S. albofasciatus*; SM, *S. marmoratus*
